# Effective Biotransformation of Variety of Guaiacyl Lignin Monomers Into Vanillin by *Bacillus pumilus*

**DOI:** 10.3389/fmicb.2022.901690

**Published:** 2022-05-11

**Authors:** Kangjia Zuo, Huanan Li, Jianhui Chen, Qiuping Ran, Mengtian Huang, Xinxin Cui, Lili He, Jiashu Liu, Zhengbing Jiang

**Affiliations:** ^1^State Key Laboratory of Biocatalysis and Enzyme Engineering, Hubei University, Wuhan, China; ^2^Hubei Key Laboratory of Industrial Biotechnology, School of Life Sciences, Hubei University, Wuhan, China

**Keywords:** vanillin, biotransformation, monooxygenase, bio-oil, *Bacillus pumilus*

## Abstract

Biotransformation has gained increasing attention due to its being an eco-friendly way for the production of value-added chemicals. The present study aimed to assess the potential of *Bacillus pumilus* ZB1 on guaiacyl lignin monomers biotransformation for the production of vanillin. Consequently, isoeugenol, eugenol, and vanillyl alcohol could be transformed into vanillin by *B. pumilus* ZB1. Based on the structural alteration of masson pine and the increase of total phenol content in the supernatant, *B. pumilus* ZB1 exhibited potential in lignin depolymerization and valorization using masson pine as the substrate. As the precursors of vanillin, 61.1% of isoeugenol and eugenol in pyrolyzed bio-oil derived from masson pine could be transformed into vanillin by *B. pumilus* ZB1. Four monooxygenases with high specific activity were identified that were involved in the transformation process. Thus, *B. pumilus* ZB1 could emerge as a candidate in the biosynthesis of vanillin by using wide guaiacyl precursors as the substrates.

## Introduction

Vanillin (4-hydroxy-3-methoxybenzaldehyde) has been widely used in many industries, such as pharmaceuticals, foods, and cosmetics (Fache et al., 2016). In recent years, the demand for vanillin in the global market increases with a stable growth rate. Chemical synthesis and natural extraction are the two common strategies to produce vanillin (Gallage and Møller, 2015). The extraction of vanillin from vanilla beans is much more expensive than the synthesis of vanillin (Gallage and Møller, 2015). To date, 20,000 tons of vanillin are produced annually and most of the vanillin is produced by a synthetic approach (Gallage and Møller, 2015; Fache et al., 2016). Chemically synthesis of vanillin from guaiacyl lignin precursors catalyzed by transition metal catalysts is more effective than the natural extraction (Augugliaro et al., 2012). However, the chemical synthesis of vanillin generally relies on fossil resources. The consumption of non-renewable resources in chemical synthesis limits the development of chemical synthesis in vanillin production (Klaus et al., 2019; Khwanjaisakun et al., 2020). Therefore, the exploitation of an alternative strategy for vanillin production using unrestricted renewable raw materials is needed.

Lignocellulose is the most abundant natural macromolecule which is considered a potential resource for the sustainable production of fuels and chemicals. Burning and direct discard of lignocellulose is a waste of resources and even causes environmental pollution (Xu et al., 2019). In general, lignin is composed of three major monomers, including guaiacyl, syringyl, and p-hydroxyphenyl units (Morya et al., 2021). The biological funneling of lignin and its derivatives is an attractive approach in lignocellulosic biorefineries because it overcomes the structural heterogeneity of lignin and selectively generates target chemicals (Beckham et al., 2016). Currently, approximately 20% of vanillin is produced by using lignin as the substrate (Khwanjaisakun et al., 2020). The industrial approach for vanillin production from lignin starts with the sulfite pulping of wood. The lignin from the sulfite pulping industry is the sole source for industrial-scale production of vanillin (Fache et al., 2016). The harsh chemical oxidation process is needed in the industrial-scale production of vanillin (Gallage and Møller, 2015). Moreover, the extraction and purification of vanillin generally need toxic organic solvents, such as benzene and toluene (Khwanjaisakun et al., 2020). Therefore, an environmentally-friendly strategy for vanillin production needs to be explored.

In nature, microorganisms are well known to be capable of degrading lignocellulose and transforming depolymerized intermediates, which gains a great interest in lignin biotransformation (Xu et al., 2019). Biodegradation of lignin has been studied primarily in fungi and bacteria (Linger et al., 2014). Among them, bacteria and their enzymes have attracted considerable attention due to the environmental adaptability that may be suitable for industrial applications. Previous studies show that bacteria can transform lignin into high value-added products such as vanillin, furfural, lipids (Sainsbury et al., 2013; Reshmy et al., 2022). The inherent structural heterogeneity of lignin and lignin depolymerized monomers brings the challenge in the utilization of lignin by microbes. Therefore, the exploitation of bacteria with lignin biotransformation potential and elucidation of related enzymatic reactions is significant for the development of green chemistry.

Guaiacyl lignin monomers are predominant units of lignin in softwood (Xu et al., 2019). Previous researches reveal that the use of guaiacyl lignin monomers as the precursors for vanillin biosynthesis is feasible (Shimoni et al., 2000; Gallage and Møller, 2015; Galadima et al., 2020). Many studies pay more attention to using one specific precursor for the biosynthesis of vanillin (Ashengroph and Amini, 2017; Yang et al., 2017; Singh et al., 2019). Nevertheless, the catalytic potential of one specific microbe on multiple substrates to generate unique value-added chemicals receives relatively less attention. Previous studies show that the *Bacillus* genus exhibits lignin degradation ability and potential in lignin valorization (Zhu et al., 2017, 2021; Mei et al., 2020). Herein, we aimed to assess the biotransformation potential of *Bacillus pumilus* ZB1 in vanillin production using different guaiacyl lignin monomers as the substrate. The potential of *B. pumilus* ZB1 in masson pine degradation was further investigated, as well as the valorization ability of bio-oil pyrolysis from masson pine.

## Materials and Methods

### Strain, Media, and Culture Conditions

*Bacillus pumilus* ZB1 (CCTCC AB2013116) was obtained from China Center for Type Culture Collection and preserved in our laboratory. *B. pumilus* ZB1 was pre-cultured in 250 mL Erlenmeyer flasks containing 50 mL of LB medium. The incubation proceeded for 12 h at 37°C with shaking at 150 rpm. Then *B. pumilus* ZB1 was inoculated into the 250 mL Erlenmeyer flasks containing 50 mL of the basal medium with the inoculum size of 5% (v/v) under the same culture conditions. The basal medium contains 5 g/L yeast extract, 5 g/L peptone, 5 g/L KNO_3_, 1 g/L KH_2_PO_4_, and 0.1 g/L MgSO_4_⋅7H_2_O (Nagar et al., 2012).

### Bioconversion Study of Guaiacyl Lignin Monomers Into Vanillin by *B. pumilus* ZB1

Bioconversion analysis of guaiacyl lignin monomers to vanillin was performed based on the actively growing 24 h culture of *B. pumilus* ZB1 in the basal medium. Isoeugenol, eugenol, and vanillyl alcohol were subsequently added to the bacterial culture with a final concentration of 1 g/L, respectively. The bioconversion experiments were carried out under different parameters, including temperature (28, 37, and 45°C), shaking speed (100, 150, and 200 rpm), pH (5.0, 7.0, and 9.0), and substrate concentration (0.5, 1, and 2 g/L). The samples were withdrawn from the bacterial culture on day 4. The biotransformation process was stopped by adding three volumes of acetonitrile into the samples. The products analysis was performed by using Primaide HPLC systems (Hitachi) equipped with an Eclipse XDB-C18 column (4.6 mm × 250 mm, 5 μm). The analytical methods were carried out as previously reported (Yamada et al., 2007). The injection volume was 10 μL. The detection wavelength was 280 nm.

GC/MS (GCMS-QP2010 Ultra, Shimadzu, Japan) equipped with an HP-5 column was used to identify the metabolites of three guaiacyl lignin monomers by *B. pumilus* ZB1. The samples were collected on day 6 and then acidified with 6 M HCl to pH 2.0. The transformed products were extracted with the equivalent volume of ethyl acetate three times. After complete dehydration with anhydrous sodium sulfate, the samples were concentrated by rotary evaporation. The concentrated samples were re-dissolved in dichloromethane. The derivatization was conducted at 60°C for 15 min. The derivatization mixture was composed of 500 μL concentrated sample, 100 μL *N*-methyl-*N*-(trimethylsilyl) trifluoroacetamide, and 100 μL pyridine. Helium was used as the carrier gas. The flow rate was 1 mL/min. The temperature program of the GC analysis was set as follows: 60°C for 3 min, followed by 5°C/min to 300°C, and then held at 300°C for 2 min. The mass spectrometry was performed with a scan range of 30–550 *m/z* (70 eV EI). The products were identified based on the NIST11 database.

### Biotransformation Study of Bio-Oil From Masson Pine by *B. pumilus* ZB1

#### Characterization of Masson Pine With *B. pumilus* ZB1 Treatment

The capacity of *B. pumilus* ZB1 on lignin biotransformation was preliminarily evaluated. *B. pumilus* ZB1 was cultured in the basal medium for 24 h. 5% of seed culture was transferred into the fresh basal medium containing 20 g/L masson pine (20–80 mesh) and incubated at 37°C with shaking at 150 rpm. The masson pine was collected after bacterial treatment for 5 days. The untreated masson pine was set as the control. The supernatant was withdrawn periodically and used for the measurement of the concentration of total phenol and reducing sugar. The determination of total phenol and reducing sugar were conducted as previously described (Yu et al., 2009; Zhang et al., 2021). Qualitative analysis of the depolymerized monomers in the supernatant was performed based on the GC/MS analysis described above. Meanwhile, the treated and untreated lignocellulosic samples were completely dried in the oven at 60°C. The surface morphology of masson pine was studied by scanning electron microscope (SEM) (JSM-6510LV, JEOL, Japan). Fourier-transform infrared (FTIR) spectroscopy analysis was performed using Nicolet iS10 FTIR spectrometer (Thermo Scientific, United States). The sample was prepared by mixing the masson pine samples and potassium bromide. The wavenumbers of softwood were identified as previously described (Pandey, 1999).

#### Characterization and Preparation of Bio-Oil by Fast Pyrolysis of Masson Pine

To characterize the bio-oil, fast pyrolysis-GC/MS was performed using Agilent 6890N GC equipped with a mass spectrometer (5975). The samples were ramped at 10°C/ms to the final temperature of 400°C and held for 25 s. The pyrolysis volatiles were further analyzed using GC. The initial temperature of GC program was 40°C. After being maintained at 40°C for 1 min, the temperature was increased by 10°C/min to 150°C and maintained at 150°C for 1 min. Thereafter, the temperature was increased by 5°C/min to 300°C and maintained at 300°C for 3 min. The split ratio was 50:1. The pyrolyzed products in bio-oil were identified based on the NIST11 database.

To prepare the bio-oil for the assessment of the potential of *B. pumilus* ZB1 in biotransformation, fast pyrolysis of masson pine particle (<0.18 mm) was performed using a Carbolite Gero TF1 Tube Furnace. Masson pine was ground and passed through the 80-mesh screen. The fast pyrolysis parameters were set as previously reported with a slight modification (Wang et al., 2018). Briefly, the samples were heated at 10°C/ms to 400°C and held for 30 min. The liquid products were collected for biotransformation analysis.

#### Biotransformation Analysis of Bio-Oil by *B. pumilus* ZB1

Based on the actively growing *B. pumilus* ZB1 culture in the basal medium as described above, 1, 5, and 10 g/L of bio-oil were added into the culture, respectively. The incubation was proceeded at 37°C with shaking at 150 rpm and the samples were withdrawn periodically. The growth of *B. pumilus* ZB1 in the basal medium containing bio-oil was measured each 24 h via UV–Vis spectrophotometer (AOE Instruments, Shanghai). The quantitative analysis of isoeugenol, eugenol, and vanillin was performed using HPLC systems as described above.

### Investigation of the Biological Process in Vanillin Biosynthesis by *B. pumilus* ZB1 Using Isoeugenol as the Model

#### Cloning of the Coding Sequences of Monooxygenases of *B. pumilus* ZB1

To verify the biotransformation capacity of *B. pumilus* ZB1 from isoeugenol to vanillin, a total of 7 monooxygenase genes annotated in the genome of *B. pumilus* were obtained. These monooxygenases were named M1–M7. The information of these genes were listed in Supplementary Table 1. The sequences of 7 monooxygenase genes were cloned into *Nde*I and *Xho*I co-digested pET28a(+), respectively. The positive recombinant plasmids were obtained by sequencing.

#### Expression and Purification

The recombinant plasmids and the negative control pET28a(+) were transformed into *Escherichia coli* BL21 (DE3), respectively. The transformants were inoculated in Luria-Bertani (LB) medium containing 50 μg/mL of kanamycin. The incubation was then conducted at 37°C with a shaking of 200 rpm overnight. The seed cultures were transferred into the fresh LB medium containing 50 μg/mL of kanamycin with 2% of inoculum size. IPTG was added into the cultures with a final concentration of 0.5 mM when OD_600_ reached approximately 0.6 to 0.8. The cultivation was continued at 25°C with shaking at 150 rpm for 12 h. Thereafter, the pellets were collected after centrifugation and then were resuspended in a one-twentieth volume of lysis buffer (50 mM Tris-HCl, 10 mM imidazole, 0.3 M NaCl, 3 mg/mL lysozyme, and 1 mM PMSF, pH 7.0). The disruption of pellets was performed using an ultrasonic processor. The cell-free extracts were prepared for isoeugenol monooxygenase activity measurement and purification. The purification of monooxygenases was conducted as previously described with a slight modification (Liu et al., 2021). Briefly, monooxygenases were eluted by elution buffer containing 500 mM of imidazole. The protein concentration was estimated using the Bradford reagent.

#### Functional Analysis of Monooxygenases From *B. pumilus* ZB1

The function of isoeugenol monooxygenase was verified using isoeugenol as the substrate for vanillin production. The reaction mixture in a total volume of 10 mL was composed of 7.5 mM isoeugenol and 10 mL cell-free extracts of recombinant *E. coli*. These reactions proceeded at 25°C, 150 rpm for 1 h. Qualitative analysis of the transformed products was carried out by GC/MS as described above.

The purified isoeugenol monooxygenase was used for the measurement of enzymatic activity. The reaction mixture was composed of 0.01 mg/mL purified isoeugenol monooxygenase and 37.2 mM isoeugenol in 50 mM Tris-HCl, pH 7.0. One unit of isoeugenol monooxygenase activity is equivalent to the amount of enzyme that converted 1 μg of isoeugenol per minute. To investigate the performance of different isoeugenol monooxygenase on vanillin production, the same reactions were conducted at different pH values. The yield of vanillin was measured using HPLC systems as described above.

#### Homology Modeling of Monooxygenase

Homology modeling of M5 was predicted using SWISS-MODEL server^1^. The template for this model was the 2.0-Å crystal structure of *E. coli* putative monooxygenase (PDB ID: 2HIQ). The generated model of M5 shared 46% sequence identity with the template.

## Results

### Biotransformation Analysis of Guaiacyl Lignin Monomers Into Vanillin by *B. pumilus* ZB1

The bioconversion potential of *B. pumilus* ZB1 on guaiacyl lignin monomers under different culture conditions was investigated (Table 1). With the incubation at different temperatures, isoeugenol, and vanillyl alcohol could be transformed into vanillin effectively at 28°C. A slight increase of vanillin production was observed using eugenol as the precursor when the biotransformation proceeded at 45°C. The change in shaking rates also influenced the yield of vanillin. The use of isoeugenol, eugenol, and vanillyl alcohol as the substrate yielded more vanillin at 100 rpm. A range of pH from 5.0 to 9.0 was used to assess the effect of pH on biotransformation. Alkaline condition showed an improvement for vanillin production from each guaiacyl lignin compound by *B. pumilus* ZB1. Whether increasing the substrate concentration could improve vanillin production was also investigated. As a result, a higher yield of vanillin was observed with the increase of the substrate concentration to 2 g/L. Overall, vanillyl alcohol could be completely transformed into vanillin by *B. pumilus* ZB1 with a low shaking rate and alkaline condition. Low shaking rate, 28°C, and alkaline pH might benefit vanillin production when using isoeugenol as the substrate. The transformation efficiency of eugenol to vanillin was relatively low in comparison with the other two precursors due to less than 1% of the substrate could be converted into vanillin.

**TABLE 1:** Yield of vanillin (mg/L) transformed by *Bacillus pumilus* ZB1 with the different guaiacyl lignin monomers as the substrate.

Parameters	Substrates		
	Isoeugenol	Eugenol	Vanilla alcohol
28°C	160.9 ± 33.92	6.1 ± 0.01	936.3 ± 78.7
37°C	103.5 ± 0.56	6 ± 0.26	557.3 ± 27.65
45°C	83.22 ± 0.74	6.7 ± 0.37	72.3 ± 10.8
100 rpm	174 ± 58	8.6 ± 1.95	1112.5 ± 73.7
150 rpm	103.5 ± 0.56	6 ± 0.26	557.3 ± 27.65
200 rpm	108 ± 26.09	6.2 ± 0.06	827 ± 71.93
pH 5.0	100.1 ± 22.88	6 ± 0.14	894.6 ± 80.3
pH 7.0	103.5 ± 0.56	6 ± 0.26	557.3 ± 27.65
pH 9.0	148.2 ± 11.71	6.4 ± 0.44	1058.8 ± 2.2
0.5 g/L	66.2 ± 0.15	6.4 ± 0.27	425.8 ± 78.93
1 g/L	103.5 ± 0.56	6 ± 0.26	557.3 ± 27.65
2 g/L	135 ± 48.96	7.8 ± 0.71	890 ± 97.43

GC/MS analysis was used for metabolites determination by using different guaiacyl lignin monomers as the substrate. The culture was conducted at pH 7.0 and 37°C with a shaking rate of 150 rpm. The result was shown in Supplementary Table 2. Vanillyl alcohol was disappeared on day 6, vanillin was still observed in the result of isoeugenol as substrate on day 6. In the results of eugenol, the isoeugenol was detected. Lactic acid was detected in all three results of metabolites, implying that *B. pumilus* ZB1 might utilize different aromatic monomers for supporting their growth. Overall, the *B. pumilus* ZB1 could convert isoeugenol, eugenol, and vanillyl alcohol into vanillin within 4 days before further biotransformation.

### Assessment of the Potential of *B. pumilus* ZB1 in Masson Pine Degradation

**FIGURE 1:**
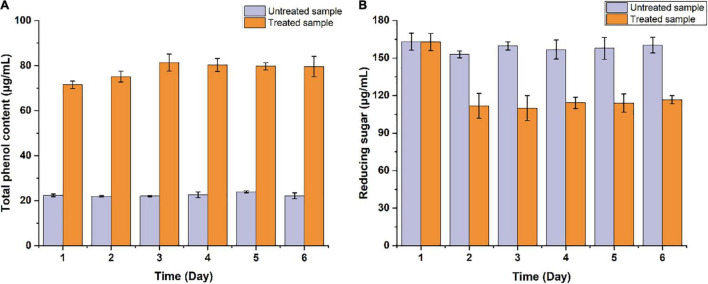
Measurement of the concentration of **(A)** total phenol and **(B)** reducing sugar in the supernatant of *Bacillus pumilus* ZB1 treated and untreated basal medium containing masson pine.

The potential of *B. pumilus* ZB1 in softwood degradation was assessed using masson pine as the substrate. As shown in Figure 1, the content of total phenol and reducing sugar in the supernatant of the bacterial treated sample significantly changed compared with the untreated sample. The total phenol content in the bacterial treated sample increased up to 71.46 μg/mL within 1 day, while in the untreated sample was 22.3 μg/mL. The reducing sugar content in the bacterial treated and the untreated samples were 111.87 and 152.94 μg/mL on day 2, respectively. In the supernatant, the total phenol content increased by 3.2-fold compared with the untreated sample, the reducing sugar decreased by 1.37-fold. Thereafter, the depolymerized products of masson pine in the supernatant were analyzed via GC/MS. As shown in Supplementary Figure 1, two guaiacyl lignin monomers were detected, including isoeugenol and vanillic acid. These results implied that *B. pumilus* ZB1 could depolymerize masson pine and further utilize isoeugenol as the precursor for vanillin production.

The effect of *B. pumilus* ZB1 treatment on the structural alteration of masson pine was further investigated. Due to the regular, smooth, and densified surface of masson pine without *B. pumilus* ZB1 treatment was observed, the internal structure of masson pine was unexposed (Supplementary Figure 2A). However, the SEM image revealed that the significant structural alterations of masson pine occurred with *B. pumilus* ZB1 treated for 5 days. The surface of masson pine appeared irregular and the internal structure was exposed together with the formation of pores (Supplementary Figure 2B). As shown in Supplementary Figure 3, FTIR spectra of the original and the treated samples confirmed the capacity of *B. pumilus* ZB1 on lignin depolymerization of masson pine. In the fingerprint region of lignin, the signals corresponding to the hydrogen bonded O-H stretching at 3,414 cm^–1^, C-H stretching at 2,935 and 2,842 cm^–1^, and unconjugated C = O stretching at 1,714 cm^–1^ were disappeared after *B. pumilus* ZB1 treatment for 5 days. In the fingerprint region of cellulose, the bands corresponding to the conjugated C = O at 1,640 cm^–1^, the asymmetric C-H deformation at 1,430 cm^–1^, the symmetric C-H deformation at 1,372 cm^–1^, the O-H in-plane deformation at 1,336 cm^–1^, and the CH_2_ wagging at 1,318 cm^–1^ were decreased. These results indicated that the degradation of lignin and the exposure of cellulose in masson pine occurred after bacterial treatment.

### Biotransformation of Bio-Oil Into Vanillin by *B. pumilus* ZB1

In the present study, we confirmed *B. pumilus* ZB1 could effectively depolymerize masson pine, implying that *B. pumilus* ZB1 is capable of involving in lignin degradation. Pyrolysis of lignocellulosic biomass may improve the biotransformation efficiency because the recalcitrant structure of lignocellulose can be fragmented (Wang et al., 2018). Herein, we prepared the bio-oil of masson pine after fast pyrolysis. Based on the identification of the aromatic compounds pyrolyzed from masson pine, isoeugenol and eugenol were detected in the bio-oil (Table 2). This result implied that the bio-oil pyrolyzed from masson pine might serve as the substrate of guaiacyl lignin monomers transformation by *B. pumilus* ZB1 for vanillin production.

**TABLE 2:** Identification of the aromatic compounds in bio-oil pyrolyzed from masson pine via py-GC/MS.

Compounds	Retention time (min)	Peak area
Benzofuran, 2,3-dihydro-	10.624	0.38
2-methoxy-4-vinylphenol	12.07	0.89
Eugenol	12.708	0.36
Vanillin	13.397	0.81
*Trans*-isoeugenol	14.254	0.94
2-methoxy-4-propyl-phenol	14.422	0.25
6-methoxy-3-methylbenzofuran	14.792	0.73
Ethanone,1-(3-hydroxy-4-methoxyphenyl)-	14.91	0.31
4-Amino-2,3-xylenol	15.716	0.14
Phenol, 4-(ethoxymethyl)-2-methoxy-	17.935	0.28
2-propenal,3-(4-hydroxy-3-methoxyphenyl)-	19.565	2.65
1-ethyl-2-benzimidazolinone	19.632	2.92
Dibutyl phthalate	23.918	3.09
1,3-benzenedicarboxylic acid, 1,3-bis(2-ethylhexyl) ester	29.295	0.54
Bis(2-ethylhexyl) phthalate	33.783	3.55
1,4-benzenedicarboxylic acid bis(2-ethylhexyl) ester	36.656	2

**FIGURE 2:**
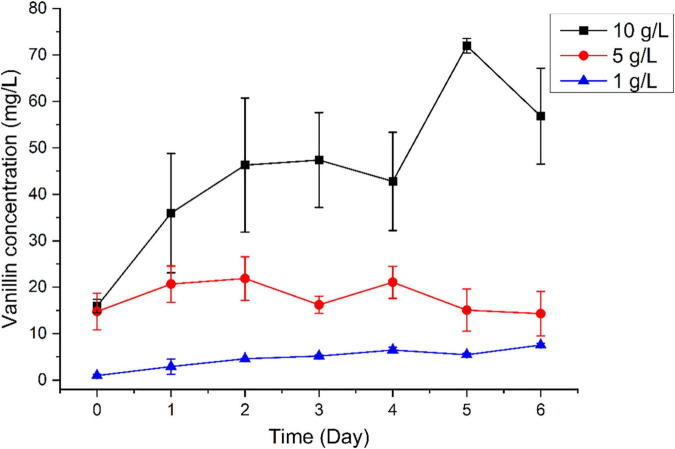
Time-course analysis of vanillin production using different concentrations of bio-oil as the substrate.

We subsequently tested the capacity of *B. pumilus* ZB1 in vanillin production using bio-oil as the substrate. Time-course analysis revealed that vanillin concentration increased along with the cultural time. Notably, when the bio-oil concentration increased, the yield of vanillin increased with the addition of a high concentration of bio-oil. As expected, with the addition of 1, 5, and 10 g/L of bio-oil into the basal medium, the concentration of vanillin increased up to 7.54, 14.31, and 56.85 mg/L, respectively, on day 6 (Figure 2). The maximal yield of vanillin was 71.99 mg/L on day 5 using 10 g/L of bio-oil as the substrate. As shown in Supplementary Table 3, the initial content of isoeugenol and eugenol in 10 g/L bio-oil was 68.3 and 23.5 mg/L, respectively. The initial content of vanillin in 10 g/L bio-oil was 15.9 mg/L. Overall, after deducting vanillin in the initial substrate, 61.1% of isoeugenol and eugenol in bio-oil was able to transform into vanillin by *B. pumilus* ZB1.

### Analysis of the Biological Process of *B. pumilus* ZB1 During Guaiacyl Lignin Biotransformation

To further characterize the biological process of *B. pumilus* ZB1 during biotransformation, the functional proteins potentially involved in the biotransformation of isoeugenol to vanillin were investigated. Based on the analysis of the annotated genome of *B. pumilus* SH-B9 (Accession number: NZ_CP011007), a total of seven monooxygenases were annotated in the genome and subsequently isolated from the genome of *B. pumilus* ZB1. Heterologous expression in *E. coli* was performed after isolation of these genes. Before the purification of monooxygenases, the enzymatic function was first evaluated using cell-free extracts of transformed *E. coli* BL21 (DE3). Based on the identification of the products using the NIST database, four out of the seven cell-free extracts exhibited isoeugenol monooxygenase activity, including M2, M3, M5, and M6 (Supplementary Figure 4). Subsequently, the recombinant monooxygenases were purified by Ni-affinity chromatography. The purity of recombinant monooxygenases was verified by SDS-PAGE (Supplementary Figure 5). Supplementary Table 4 summarized the properties of monooxygenases after purification. The specific activities of purified M2, M3, M5, and M6 were 30.82, 26.09, 29.74, and 52.16 U/mg, respectively.

**FIGURE 3:**
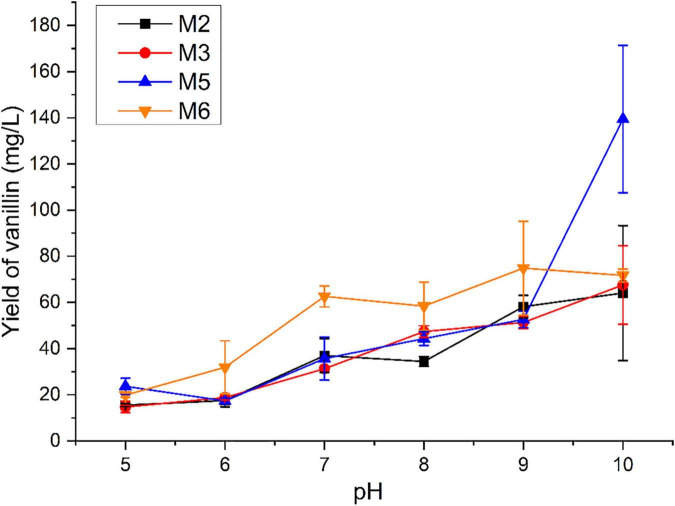
Measurement of vanillin production catalyzed by monooxygenases using isoeugenol as the substrate under different pH conditions.

The biological function of four identified monooxygenases in the isoeugenol transformation was evaluated with the pH ranging from 5 to 10. As shown in Figure 3, vanillin could be detected using isoeugenol as the substrate at a wide range of pH. The low yield of vanillin was observed under the acidic condition. Interestingly, these four monooxygenases preferred converting isoeugenol into vanillin under alkaline conditions. The maximal yield of vanillin was obtained at a pH ranging from 9 to 10. With the M2, M3, and M5 as the biocatalyst, the highest yield of vanillin increased up to 64.036, 67.539, and 139.467 mg/L, respectively, at pH 10.0. 74.838 mg/L of vanillin was detected after incubation with M6 at pH 9.0.

## Discussion

Guaiacyl units are the most abundant compounds encountered during softwood lignin degradation and depolymerization (Morya et al., 2021). As a unique chemical manufactured on the industrial scale from lignocellulosic biomass, vanillin plays an important role in practical applications, such as food, cosmetic, and pharmaceutical industries (Priefert et al., 2001). The conventional method of extracting vanillin from depolymerized lignin fragments generally requires organic solvents (Fache et al., 2016), which may increase the cost and cause environmental pollution. In our study, we found that different guaiacyl lignin monomers could be transformed into vanillin by *B. pumilus* ZB1. Theoretically, the biotransformation of different lignin monomers to vanillin might be an effective strategy in vanillin production. Previous literature proposes that vanillin can be further transformed into vanillic acid by vanillin dehydrogenase (Xu et al., 2019; Nguyen et al., 2021). In our study, vanillic acid was detected on day 4 and subsequently lactic acid was detected on day 6. This result implied that vanillin might be further catabolized by *B. pumilus* ZB1 along with the time. The deletion of vanillin dehydrogenase gene in the genome of microbes using genetic manipulation results in vanillin accumulation without undesired catabolism (Di Gioia et al., 2011; Fleige et al., 2013). The elimination of downstream catabolism of vanillin in *B. pumilus* ZB1 might be carried out in future studies. Overall, these results suggested that *B. pumilus* ZB1 has the capability in the biotransformation of different guaiacyl lignin monomers into vanillin within 4 days.

In many cases, *Bacillus* species are capable of depolymerizing lignocellulosic biomass, which raises great attention for its application in biorefinery (Lee et al., 2019; Mei et al., 2020). Bacillus species can depolymerize the lignin macromolecule into low-molecular-weight aromatic monomers. The ligninolytic enzymes originating from bacteria play a key role in lignin depolymerization (Lee et al., 2019; Mei et al., 2020). In our study, the depolymerization of masson pine was observed with the treatment of *B. pumilus* ZB1 based on the results of the measurement of the total phenol and reducing sugar in the supernatant, as well as the structural characterization. Masson pine (*Pinus massoniana* Lamb.) is a typical softwood that is predominantly composed of guaiacyl lignin (Xu et al., 2019). Masson pine is an afforested tree species in subtropical areas, which is also used for the production of pulpwood, building wood, and other raw materials (Yuan et al., 2021). Inevitably, the generation of lignocellulosic waste increases the burden to the environment. In our study, *B. pumilus* ZB1 could depolymerize masson pine and then generate guaiacyl lignin monomers. The depolymerized product, isoeugenol, might serve as the precursor of vanillin synthesis by *B. pumilus* ZB1.

Fast pyrolysis of lignocellulosic biomass is a potential way to produce bio-based chemicals. The liquid, char, and gas derived from biomass were obtained with pyrolysis techniques at 400–650°C (Dai et al., 2020). Among them, the liquid bio-oil contains a variety of value-added chemicals from lignin (Wang et al., 2020). The sources of lignocellulosic biomass and the operating conditions of pyrolysis generally influence the final composition of the products in bio-oil (Wang et al., 2019). Softwood is composed of mostly guaiacyl lignin. The composition of masson pine contributed to the complexity of the final products in bio-oil. The pyrolysis of softwood mainly produces guaiacyl lignin monomers in the bio-oil (Wang et al., 2018). It implies that the bio-oil pyrolyzed from softwood may serve as the precursor in vanillin synthesis by microbes. However, the complex composition of bio-oil is toxic to microbial activity, which leads to the insufficient utilization of bio-oil by microorganisms (Liang et al., 2013). In our study, we found that *B. pumilus* ZB1 was capable of transforming isoeugenol and eugenol into vanillin, respectively. The presence of isoeugenol and eugenol in bio-oil might benefit transform different guaiacyl monomer to produce vanillin by *B. pumilus* ZB1. Additionally, the growth of *B. pumilus* ZB1 could be improved when the bio-oil was added into the basal medium, especially increasing the concentration of bio-oil up to 10 g/L (Supplementary Figure 6). Fatty acids generally exhibit the toxicity toward the growth of microorganisms (Al-Shmgani et al., 2019). One of the typical fatty acids, n-hexadecanoic acid, was detected in the bio-oil pyrolyzed from masson pine (data not shown). The higher yield of vanillin was observed with the addition of high concentration of bio-oil. These results implied that *B. pumilus* ZB1 not only could convert different guaiacyl lignin monomers into vanillin but also show tolerance to the toxicity of bio-oil.

In prokaryotes, the biosynthesis of vanillin is a complicated pathway related to many enzymatic systems. Eugenol can be used as the precursor involving ferulic acid biosynthesis (Ryu et al., 2010), which can be converted into vanillin through non-oxidative decarboxylation and coenzyme A (CoA)-dependent non-β-oxidation in bacteria (Xu et al., 2019). Vanillyl alcohol oxidase can also produce vanillin by oxidizing of the vanillyl alcohol (van den Heuvel et al., 2001). Isoeugenol monooxygenases derived from the *Pseudomonas* species have been proven that can use isoeugenol as the precursor for vanillin biosynthesis (Yamada et al., 2007; Wang et al., 2021). *Bacillus* species are known that is capable of converting isoeugenol to vanillin, such as *Bacillus subtilis* B2, *B. subtilis* MTCC 1427, and *Bacillus fusiformis* CGMCC1347 (Shimoni et al., 2000; Zhao et al., 2006; Rana et al., 2013). The genomic sequencing of *B. pumilus* suggests that several monooxygenases may involve in the biotransformation of isoeugenol to vanillin (Su et al., 2011). Nevertheless, the biological function of monooxygenases in *B. pumilus* strain has not been characterized. We thus specifically investigated the contribution of monooxygenases to guaiacyl lignin biotransformation using isoeugenol as the model.

Heterologous expression of monooxygenases in *E. coli* BL21 (DE3) was carried out. Based on the preliminary evaluation of monooxygenase activity, four out of the seven monooxygenases could transform isoeugenol into vanillin, including M2, M3, M5, and M6. SDS-PAGE confirmed that the molecular weights of recombinant monooxygenases containing His-tag consistent with the prediction results, including M2, M3, and M6. The molecular weight of recombinant M5 was higher than that of the predicted result. Thereafter, homology modeling of M5 was conducted using the SWISS-MODEL server. As a result, M5 has formed as the homo-dimer, which resulted in a larger molecular weight. Noteworthily, the specific activity of the isoeugenol monooxygenases described in the present study was much higher than that of monooxygenases from different microorganisms as previously reported (Supplementary Table 4). *B. pumilus* is one of the rhizobacteria which shows a good adaption to the environment (Liu et al., 2021). The practical application of the enzymatic systems from *B. pumilus* generally exhibits good performance in the alkaline environment, such as laccase, feruloyl esterase, and endoxylanase (Asha Poorna and Prema, 2007; Duan et al., 2021; Liu et al., 2021). In our study, the conversion of isoeugenol to vanillin by monooxygenases in the alkaline environment was more effective than that in the acidic environment, implying that *B. pumilus* ZB1 and its enzymatic systems could keep biological activity in an extreme environment and improve lignin biotransformation for vanillin production.

## Conclusion

In this study, vanillin could be synthesized by *B. pumilus* ZB1 using different guaiacyl monomers as the substrate. The structural characterization confirmed that *B. pumilus* ZB1 could depolymerize masson pine and generate the precursor of vanillin. The pyrolysis-GC/MS suggested that the bio-oil from masson pine might consider as the source for vanillin production by *B. pumilus* ZB1. 61.1% of isoeugenol and eugenol in bio-oil could be transformed into vanillin. Four of the seven monooxygenases with high specific activity are involved in transforming isoeugenol into vanillin. Overall, *B. pumilus* ZB1 showed potential in the valorization of guaiacyl lignin for vanillin synthesis.

## Data Availability Statement

The original contributions presented in the study are included in the article/Supplementary Material; further inquiries can be directed to the corresponding authors.

## Author Contributions

KZ: conceptualization, writing – original draft, and preparation. HL: data curation and validation. JC: methodology. QR: software. MH: data curation. XC: formal analysis. LH: validation. JL: investigation. ZJ: funding acquisition. All authors contributed to the article and approved the submitted version.

## Conflict of Interest

The authors declare that the research was conducted in the absence of any commercial or financial relationships that could be construed as a potential conflict of interest.

## Publisher’s Note

All claims expressed in this article are solely those of the authors and do not necessarily represent those of their affiliated organizations, or those of the publisher, the editors and the reviewers. Any product that may be evaluated in this article, or claim that may be made by its manufacturer, is not guaranteed or endorsed by the publisher.

## Footnotes

1
https://swissmodel.expasy.org

## References

[B1] Al-Shmgani H. M. Kadri Z. Al-Halbosiy M. Dewir Y. (2019). Phytochemical analysis, cytotoxicity and antioxidant activity of cuckoo pint (Arum maculatum) leaf extract. *Acta Biol. Szegediensis.* 63 119–124. 10.14232/abs.2019.2.119-124

[B2] Asha Poorna C. Prema P. (2007). Production of cellulase-free endoxylanase from novel alkalophilic thermotolerent Bacillus pumilus by solid-state fermentation and its application in wastepaper recycling. *Bioresour. Technol.* 98 485–490. 10.1016/j.biortech.2006.02.033 16844369

[B3] Ashengroph M. Amini J. (2017). Bioconversion of isoeugenol to vanillin and vanillic acid using the resting cells of Trichosporon asahii. *3Biotech.* 7:358. 10.1007/s13205-017-0998-9 28979831 PMC5626669

[B4] Augugliaro V. Camera-Roda G. Loddo V. Palmisano G. Palmisano L. Parrino F. (2012). Synthesis of vanillin in water by TiO2 photocatalysis. *Appl. Catal. B* 111-112 555–561. 10.1016/j.apcatb.2011.11.007

[B5] Beckham G. T. Johnson C. W. Karp E. M. SalvachúA D. Vardon D. R. (2016). Opportunities and challenges in biological lignin valorization. *Curr. Opin. Biotechnol.* 42 40–53. 10.1016/j.copbio.2016.02.030 26974563

[B6] Dai L. Wang Y. Liu Y. He C. Ruan R. Yu Z. (2020). A review on selective production of value-added chemicals via catalytic pyrolysis of lignocellulosic biomass. *Sci. Total Environ.* 749:142386. 10.1016/j.scitotenv.2020.142386 33370899

[B7] Di Gioia D. Luziatelli F. Negroni A. Ficca A. G. Fava F. Ruzzi M. (2011). Metabolic engineering of Pseudomonas fluorescens for the production of vanillin from ferulic acid. *J. Biotechnol*. 156, 309–316. 10.1016/j.jbiotec.2011.08.014 21875627

[B8] Duan X. Dai Y. Zhang T. (2021). Characterization of Feruloyl Esterase from Bacillus pumilus SK52.001 and Its Application in Ferulic Acid Production from De-Starched Wheat Bran. *Foods* 10:1229. 10.3390/foods10061229 34071417 PMC8228269

[B9] Fache M. Boutevin B. Caillol S. (2016). Vanillin Production from Lignin and Its Use as a Renewable Chemical. *ACS Sustain. Chem. Eng.* 4 35–46. 10.1021/acssuschemeng.5b01344

[B10] Fleige C. Hansen G. Kroll J. Steinbüchel A. (2013). Investigation of the Amycolatopsis sp. strain ATCC 39116 vanillin dehydrogenase and its impact on the biotechnical production of vanillin. *Appl. Environ. Microbiol.* 79, 81–90. 10.1128/AEM.02358-12 23064333 PMC3536076

[B11] Galadima A. I. Salleh M. M. Hussin H. Chong C. S. Yahya A. Mohamad S. E. (2020). Biovanillin: production concepts and prevention of side product formation. *Biomass. Convers. Biorefinery.* 10 589–609. 10.1007/s13399-019-00418-0

[B12] Gallage N. J. Møller B. L. (2015). Vanillin–Bioconversion and Bioengineering of the Most Popular Plant Flavor and Its De Novo Biosynthesis in the Vanilla Orchid. *Mol. Plant* 8 40–57. 10.1016/j.molp.2014.11.008 25578271

[B13] Khwanjaisakun N. Amornraksa S. Simasatitkul L. Charoensuppanimit P. Assabumrungrat S. (2020). Techno-economic analysis of vanillin production from Kraft lignin: Feasibility study of lignin valorization. *Bioresour. Technol.* 299:122559. 10.1016/j.biortech.2019.122559 31877478

[B14] Klaus T. Seifert A. Häbe T. Nestl B. M. Hauer B. (2019). An Enzyme Cascade Synthesis of Vanillin. *Catalysts* 9:252. 10.3390/catal9030252

[B15] Lee S. Kang M. Bae J.-H. Sohn J.-H. Sung B. H. (2019). Bacterial Valorization of Lignin: Strains, Enzymes, Conversion Pathways, Biosensors, and Perspectives. *Front. Bioeng. Biotechnol.* 7:209. 10.3389/fbioe.2019.00209 31552235 PMC6733911

[B16] Liang Y. Zhao X. Chi Z. Rover M. Johnston P. Brown R. (2013). Utilization of acetic acid-rich pyrolytic bio-oil by microalga Chlamydomonas reinhardtii: Reducing bio-oil toxicity and enhancing algal toxicity tolerance. *Bioresour. Technol.* 133 500–506. 10.1016/j.biortech.2013.01.134 23455221

[B17] Linger J. G. Vardon D. R. Guarnieri M. T. Karp E. M. Hunsinger G. B. Franden M. A. (2014). Lignin valorization through integrated biological funneling and chemical catalysis. *Proc. Natl. Acad. Sci. U.S.A.* 111:12013. 10.1073/pnas.1410657111 25092344 PMC4143016

[B18] Liu J. Chen J. Zuo K. Li H. Peng F. Ran Q. (2021). Chemically induced oxidative stress improved bacterial laccase-mediated degradation and detoxification of the synthetic dyes. *Ecotoxicol. Environ. Saf.* 226:112823. 10.1016/j.ecoenv.2021.112823 34597843

[B19] Mei J. Shen X. Gang L. Xu H. Wu F. Sheng L. (2020). A novel lignin degradation bacteria-Bacillus amyloliquefaciens SL-7 used to degrade straw lignin efficiently. *Bioresour. Technol.* 310:123445. 10.1016/j.biortech.2020.123445 32361649

[B20] Morya R. Kumar M. Shekhar Thakur I. (2021). Bioconversion of syringyl lignin into malic acid by Burkholderia sp ISTR5. *Bioresour. Technol.* 330:124981. 10.1016/j.biortech.2021.124981 33756182

[B21] Nagar S. Mittal A. Kumar D. Gupta V. K. (2012). Production of alkali tolerant cellulase free xylanase in high levels by Bacillus pumilus SV-205. *Int. J. Biol. Macromol.* 50 414–420. 10.1016/j.ijbiomac.2011.12.026 22227307

[B22] Nguyen L. T. Phan D.-P. Sarwar A. Tran M. H. Lee O. K. Lee E. Y. (2021). Valorization of industrial lignin to value-added chemicals by chemical depolymerization and biological conversion. *Industrial Crop. Prod.* 161:113219. 10.1016/j.indcrop.2020.113219

[B23] Pandey K. (1999). A Study of Chemical Structure of Soft and Hardwood and Wood Polymers by Ftir Spectroscopy. *J. Appl. Polym. Sci.* 71 1969–1975. 10.1002/(sici)1097-4628(19990321)71:12<1969::aid-app6>3.0.co;2-d

[B24] Priefert H. Rabenhorst J. Steinbüchel A. (2001). Biotechnological production of vanillin. *Appl. Microbiol. Biotechnol.* 56 296–314. 10.1007/s002530100687 11548997

[B25] Rana R. Mathur A. Jain C. Sharma S. Mathur G. (2013). Microbial production of vanillin. *Int. J. Biotechnol. Bioeng. Res.* 4 227–234. 10.1590/S1517-83822010000300001 24031526 PMC3768639

[B26] Reshmy R. Athiyaman Balakumaran P. Divakar K. Philip E. Madhavan A. Pugazhendhi A. (2022). Microbial valorization of lignin: prospects and challenges. *Bioresour. Technol*. 344:126240. 10.1016/j.biortech.2021.126240 34737164

[B27] Ryu J.-Y. Seo J. Unno T. Ahn J.-H. Yan T. Sadowsky M. J. (2010). Isoeugenol monooxygenase and its putative regulatory gene are located in the eugenol metabolic gene cluster in *Pseudomonas nitroreducens* Jin1. *Arch. Microbiol.* 192 201–209. 10.1007/s00203-010-0547-y 20091296

[B28] Sainsbury P. D. Hardiman E. M. Ahmad M. Otani H. Seghezzi N. Eltis L. D. (2013). Breaking down lignin to high-value chemicals: the conversion of lignocellulose to vanillin in a gene deletion mutant of Rhodococcus jostii RHA1. *ACS Chem. Biol*. 8, 2151–2156. 10.1021/cb400505a 23898824

[B29] Shimoni E. Ravid U. Shoham Y. (2000). Isolation of a Bacillus sp. capable of transforming isoeugenol to vanillin. *J. Biotechnol.* 78 1–9. 10.1016/s0168-1656(99)00199-6 10702906

[B30] Singh A. Mukhopadhyay K. Ghosh Sachan S. (2019). Biotransformation of eugenol to vanillin by a novel strain Bacillus safensis SMS1003. *Biocatal. Biotransform.* 37 291–303. 10.1080/10242422.2018.1544245

[B31] Su F. Hua D. Zhang Z. Wang X. Tang H. Tao F. (2011). Genome Sequence of Bacillus pumilus S-1, an Efficient Isoeugenol-Utilizing Producer for Natural Vanillin. *J. Bacteriol.* 193 6400–6401. 10.1128/JB.06170-11 22038964 PMC3209209

[B32] van den Heuvel R. H. H. Fraaije M. W. Laane C. Van Berkel W. J. H. (2001). Enzymatic Synthesis of Vanillin. *J. Agric. Food. Chem.* 49 2954–2958. 10.1021/jf010093j 11409992

[B33] Wang L. Li J. Chen Y. Yang H. Shao J. Zhang X. (2019). Investigation of the pyrolysis characteristics of guaiacol lignin using combined Py-GC × GC/TOF-MS and in-situ FTIR. *Fuel* 251 496–505.

[B34] Wang L. Zhang R. Li J. Guo L. Yang H. Ma F. (2018). Comparative study of the fast pyrolysis behavior of ginkgo, poplar, and wheat straw lignin at different temperatures. *Industrial Crop. Prod.* 122 465–472. 10.1016/j.indcrop.2018.06.038

[B35] Wang Q. Wu X. Lu X. He Y. Ma B. Xu Y. (2021). Efficient Biosynthesis of Vanillin from Isoeugenol by Recombinant Isoeugenol Monooxygenase from *Pseudomonas nitroreducens* Jin1. *Appl. Biochem. Biotechnol.* 193 1116–1128. 10.1007/s12010-020-03478-5 33411131

[B36] Wang Y. Han Y. Hu W. Fu D. Wang G. (2020). Analytical strategies for chemical characterization of bio-oil. *J. Sep. Sci.* 43 360–371. 10.1002/jssc.201901014 31769601

[B37] Xu Z. Lei P. Zhai R. Wen Z. Jin M. (2019). Recent advances in lignin valorization with bacterial cultures: microorganisms, metabolic pathways, and bio-products. *Biotechnol. Biofuels* 12:32. 10.1186/s13068-019-1376-0 30815030 PMC6376720

[B38] Yamada M. Okada Y. Yoshida T. Nagasawa T. (2007). Purification, characterization and gene cloning of isoeugenol-degrading enzyme from *Pseudomonas putida* Ie27. *Arch. Microbiol.* 187:511. 10.1007/s00203-007-0218-9 17516050

[B39] Yang T. X. Zhao L. Q. Wang J. Song G. L. Liu H. M. Cheng H. (2017). Improving Whole-Cell Biocatalysis by Addition of Deep Eutectic Solvents and Natural Deep Eutectic Solvents. *Acs Sustain. Chem. Eng.* 5 5713–5722. 10.1021/acssuschemeng.7b00285

[B40] Yu H. Guo G. Zhang X. Yan K. Xu C. (2009). The effect of biological pretreatment with the selective white-rot fungus Echinodontium taxodii on enzymatic hydrolysis of softwoods and hardwoods. *Bioresour. Technol.* 100 5170–5175. 10.1016/j.biortech.2009.05.049 19545999

[B41] Yuan C. Zhang Z. Jin G. Zheng Y. Zhou Z. Sun L. (2021). Genetic parameters and genotype by environment interactions influencing growth and productivity in Masson pine in east and central China. *Forest Ecol. Manag.* 487:118991. 10.1016/j.foreco.2021.118991

[B42] Zhang R. Wang L. Shi C. Shi Q. Ma F. Zhang X. (2021). Structural Characterization of Lignin-Carbohydrate Complexes (LCCS) and Their Biotransformation by Intestinal Microbiota In Vitro. *J. Agric. Food. Chem.* 69 12880–12890. 10.1021/acs.jafc.1c03519 34634902

[B43] Zhao L.-Q. Sun Z.-H. Zheng P. He J.-Y. (2006). Biotransformation of isoeugenol to vanillin by Bacillus fusiformis Cgmcc1347 with the addition of resin HD-8. *Proc. Biochem.* 41 1673–1676. 10.1016/j.procbio.2006.02.007

[B44] Zhu D. Xu L. Sethupathy S. Si H. Ahmad F. Zhang R. (2021). Decoding lignin valorization pathways in the extremophilic Bacillus ligniniphilus L1 for vanillin biosynthesis. *Green. Chem.* 23 9554–9570. 10.1039/d1gc02692e

[B45] Zhu D. Zhang P. Xie C. Zhang W. Sun J. Qian W.-J. (2017). Biodegradation of alkaline lignin by Bacillus ligniniphilus L1. *Biotechnol. Biofuels* 10:44. 10.1186/s13068-017-0735-y 28239416 PMC5320714

