# Supplementary material for: Effective Biotransformation of Variety of Guaiacyl Lignin Monomers Into Vanillin by Bacillus pumilus
Source: Front Microbiol. 2022 May 11;13:901690. doi: 10.3389/fmicb.2022.901690 (PMC9130762; doi:10.3389/fmicb.2022.901690)
Supplement: Supplementary file 1 [file Data_Sheet_1.docx]

Supplementary materials

**Effective biotransformation of variety of guaiacyl lignin monomers into vanillin by *Bacillus pumilus***

Kangjia Zuo^1,2^, Huanan Li^1,2^, Jianhui Chen^1,2^, Qiuping Ran^1,2^, Mengtian Huang^1,2^, Xinxin Cui^1,2^, Lili He^1,2^, Jiashu Liu^1,2,*^, Zhengbing Jiang^1,2,*^

^1^*State Key Laboratory of Biocatalysis and Enzyme Engineering, Hubei University, Wuhan, 430062, P.R. China*

^2^*Hubei Key Laboratory of Industrial Biotechnology, School of Life Science, Hubei University, Wuhan 430062, P.R. China*

* Corresponding authors

E-mail: [jsliu@hubu.edu.cn](mailto:jsliu@hubu.edu.cn) (Jiashu Liu)

E-mail: [zhbjiang@hubu.edu.cn](mailto:zhbjiang@hubu.edu.cn) (Zhengbing Jiang)


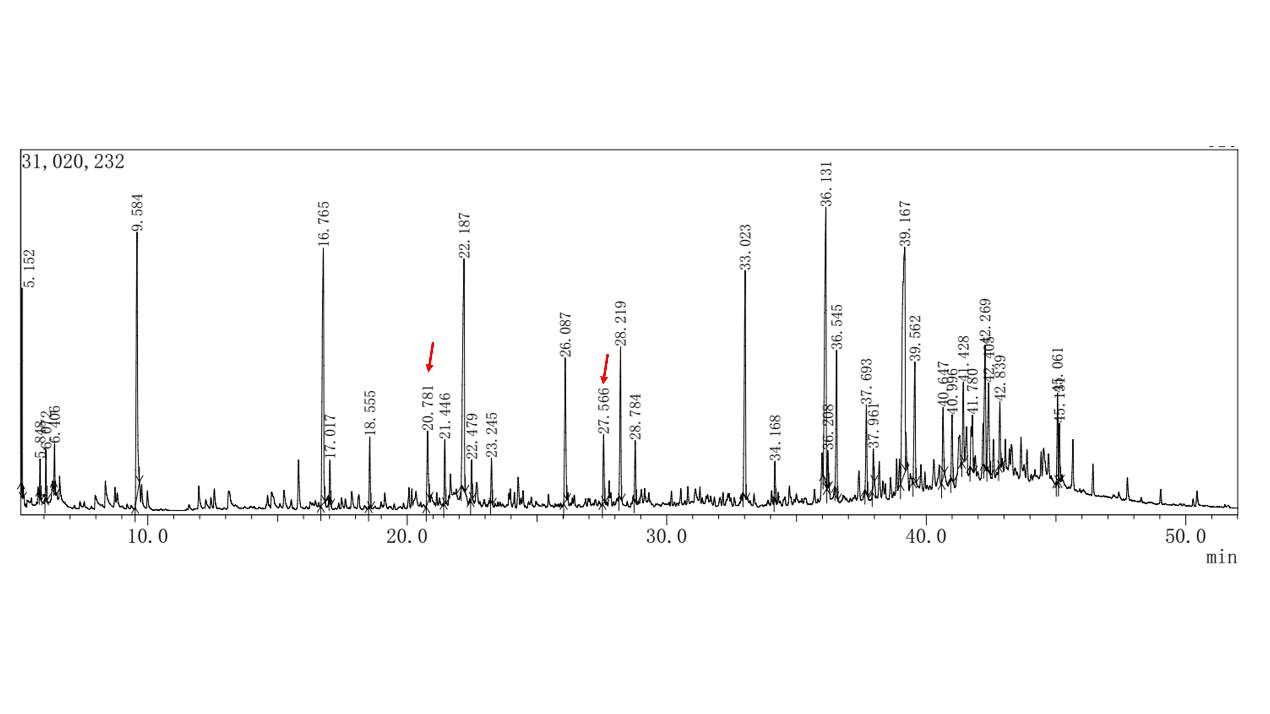


**Figure S1** GC/MS analysis of depolymerized products of masson pine in the supernatant after *B. pumilus* ZB1 treatment for 4 days. The red arrow represents the identified products: isoeugenol (20.78 min) and vanillic acid (27.566 min)


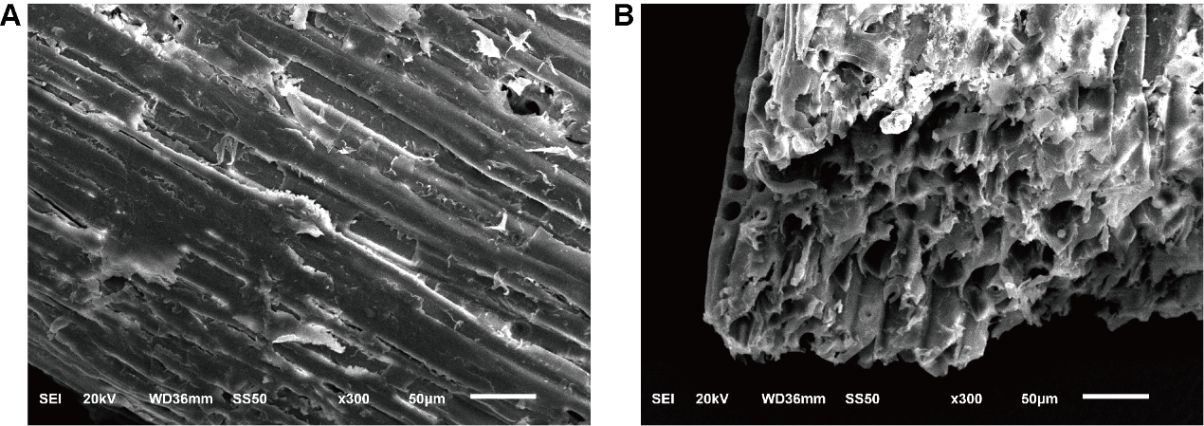


**Figure S2** SEM images of untreated masson pine control (A) and masson pine treated with *B. pumilus* ZB1 for 5 days (B)


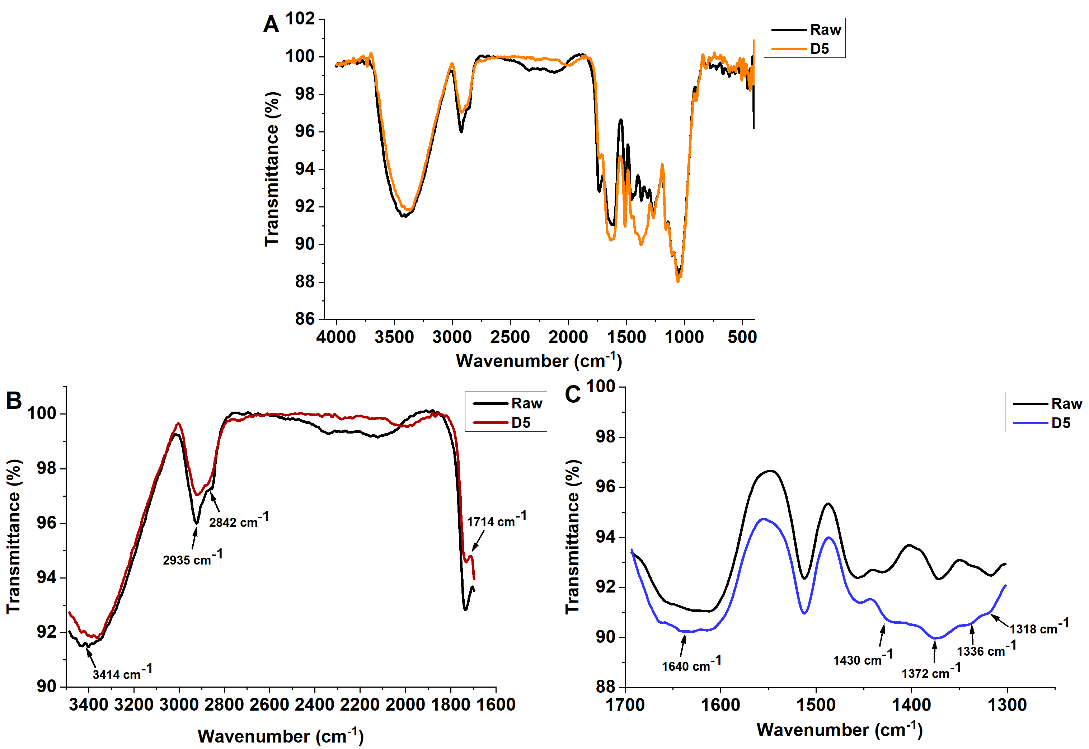


**Figure S3** FTIR spectra of masson pine treated with *B. pumilus* ZB1 for 5 days. The raw masson pine was set as the control. (A) overview of the fingerprint region of masson pine (B) the fingerprint region of lignin (C) the fingerprint region of cellulose


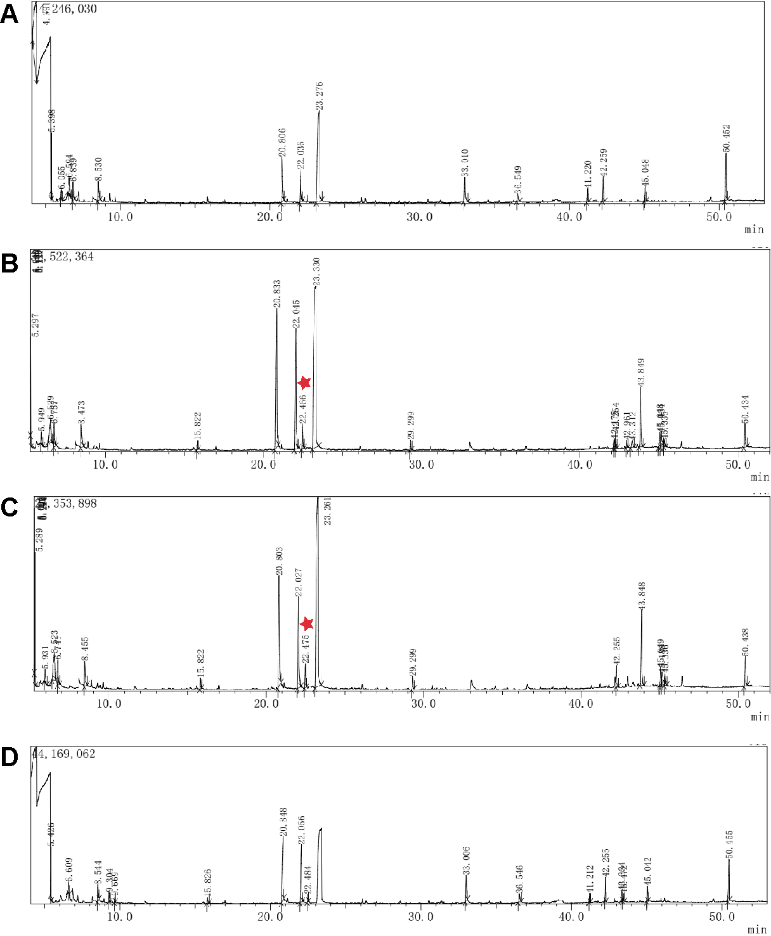


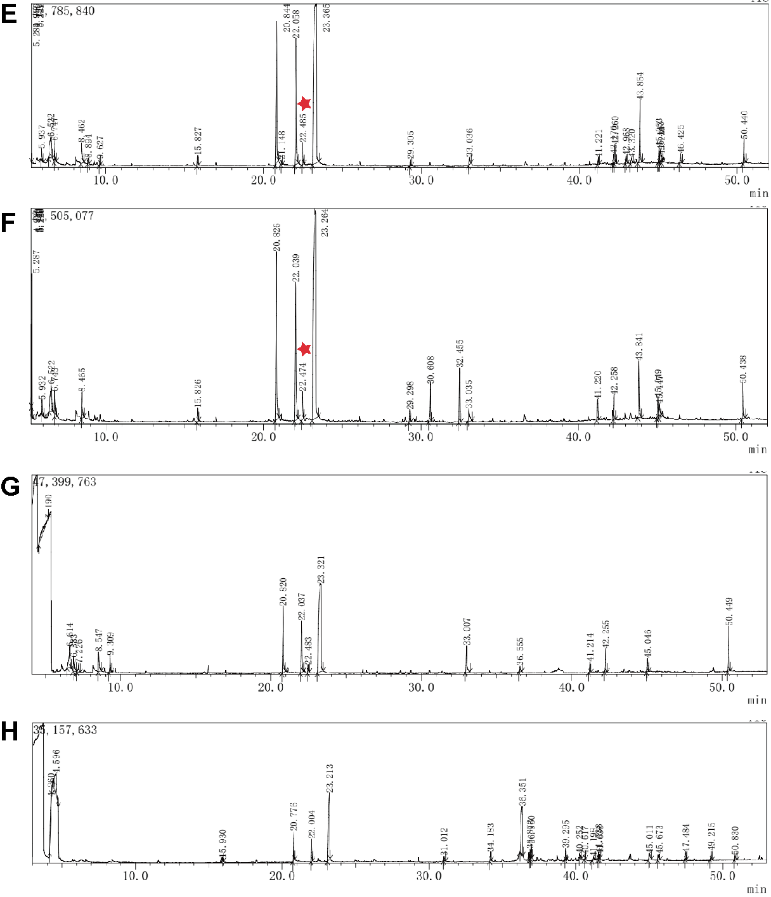


**Figure S4** Functional analyses of monooxygenases involved in the biotransformation of isoeugenol to vanillin via GC/MS analysis. (A) M1; (B) M2; (C) M3; (D) M4; (E) M5; (F) M6; (G) M7; (H) cell-free extract of recombinant *E. coli* transformed by the empty vector pET28a(+). The red star represents vanillin was detected


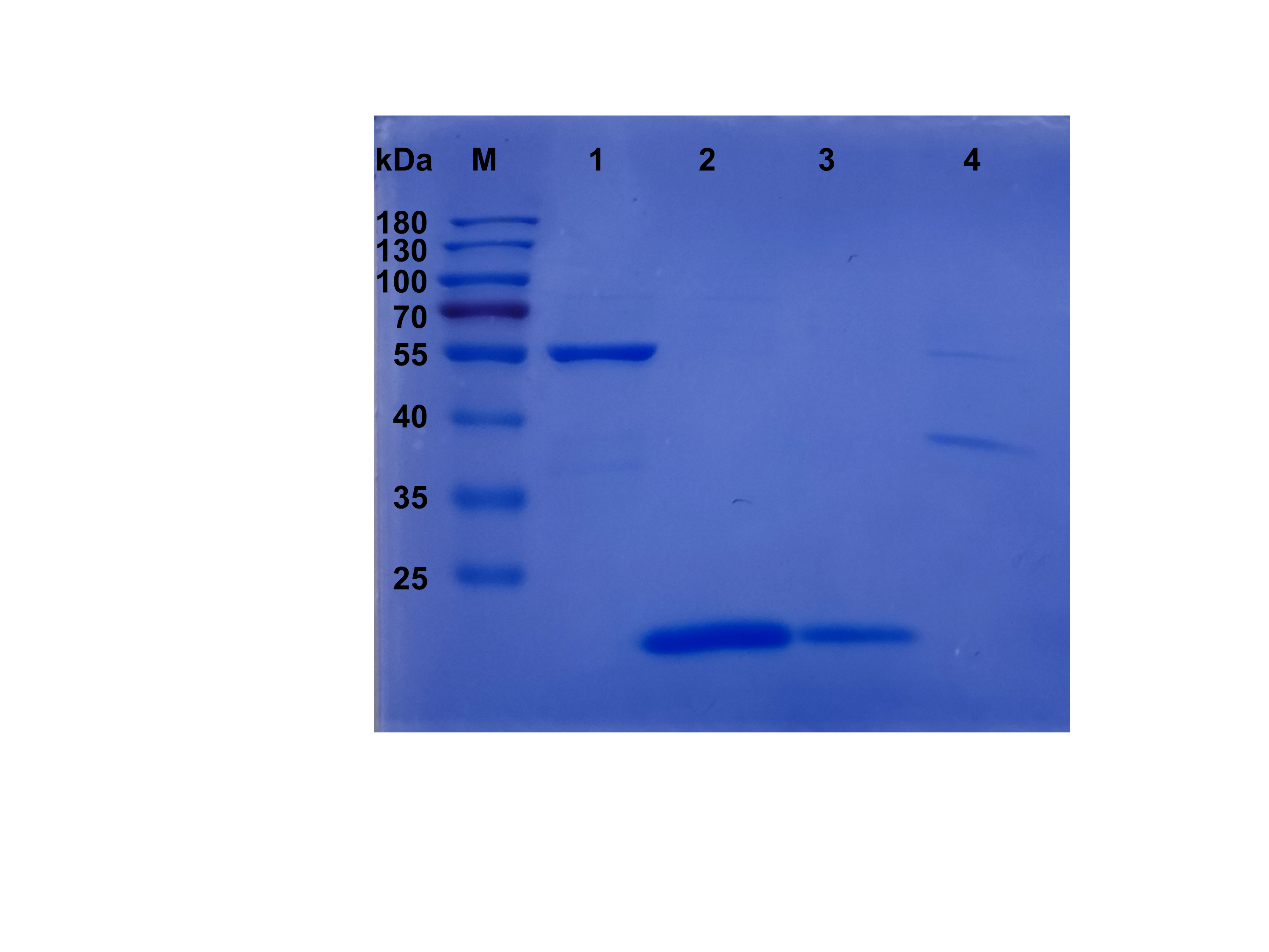


**Figure S5** Electrophoretic analysis of purified monooxygenases by SDS-PAGE. Lane M: Protein marker; Lane 1: purified M2; Lane 2: purified M3; Lane 3: purified M5; Lane 4: purified M6


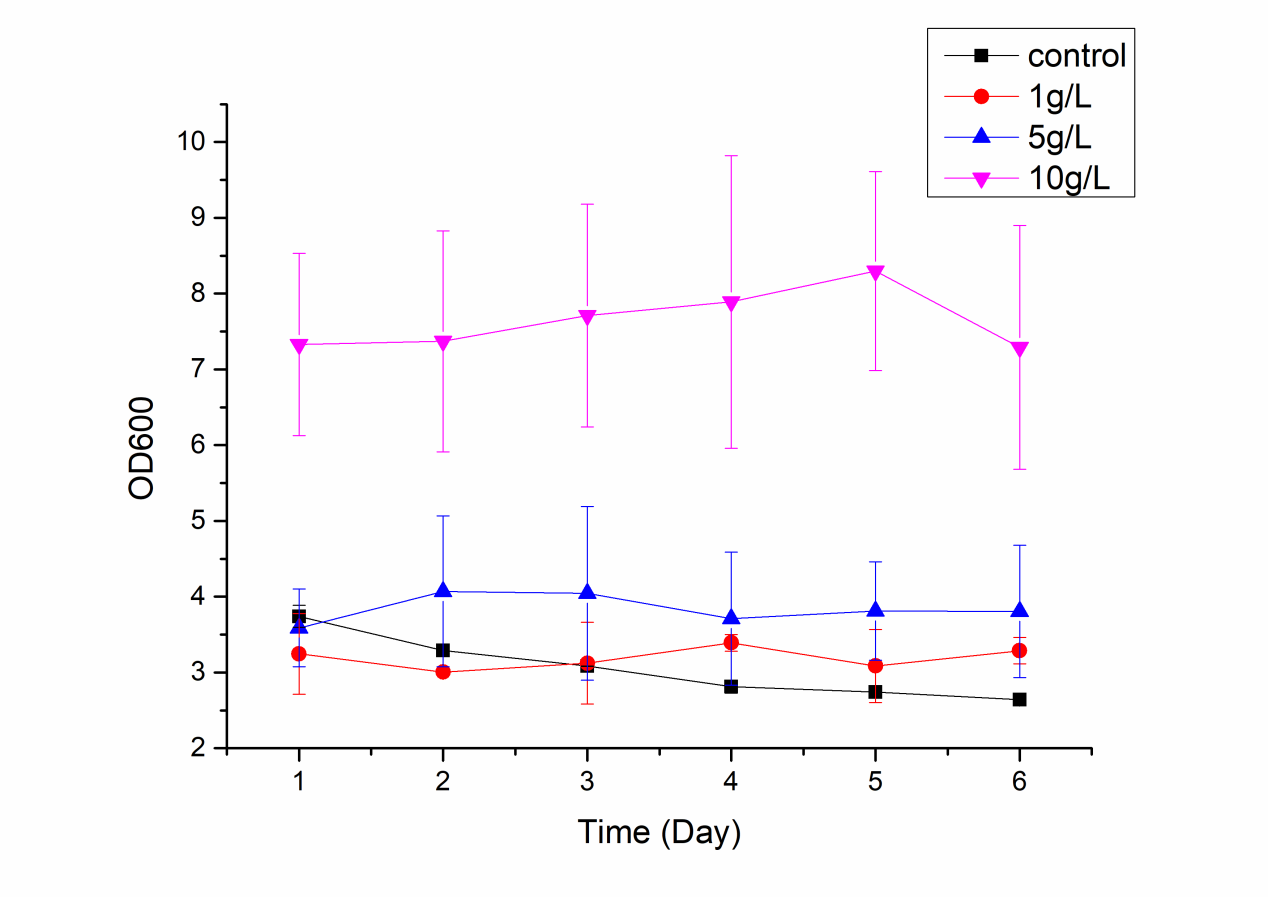


**Figure S6** The measurement of the bacterial growth with the addition of bio-oil in different concentrations

**Table S1** Monooxygenase gene information used in this study

| No. | Annotation | Sequence length (bp) | Molecular weight (kDa) | GenBank accession No. |
| --- | --- | --- | --- | --- |
| M1 | FAD-dependent monooxygenase | 1134 | 43.4 | OK317388 |
| M2 | SidA/IucD/PvdA family monooxygenase | 1275 | 49.1 | OK317389 |
| M3 | antibiotic biosynthesis monooxygenase | 486 | 18.7 | OK317390 |
| M4 | FAD-dependent monooxygenase | 1170 | 43.9 | OK317391 |
| M5 | monooxygenase | 312 | 11.8 | OK317392 |
| M6 | nitronate monooxygenase | 1032 | 37.3 | OK317393 |
| M7 | antibiotic biosynthesis monooxygenase | 294 | 10.9 | OK317394 |

**Table S2** Identification of the transformed metabolites of guaiacyl lignin monomers on day 6 via GC/MS

| Identified products | Substrates | | |
| --- | --- | --- | --- |
|  | Isoeugenol | Eugenol | Vanillyl alcohol |
| Vanillin | 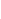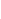＋ | － | － |
| Isoeugenol | － | 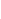＋ | － |
| Phenylacetic acid | 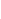＋ | － | ＋ |
| Lactic acid | 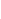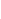＋ | 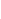＋ | ＋ |
| Phloroglucinol | － | － | ＋ |
| 2,6-di-tert-butylphenol | － | 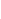＋ | － |
| 3-hydroxyphenylacetic acid | － | 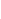＋ | － |
| Isophthalic acid | － | － | ＋ |
| 3-hydroxybenzyl alcohol | － | － | ＋ |
| 3-methoxy-4-hydroxyphenylethylene glycol | 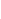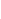＋ | － | － |
| 3-methoxy-4-hydroxyphenylglycolic acid | 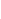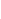＋ | － | － |

Plus (+) symbol represents the corresponding metabolite was detected. Minus (-) symbol represents the corresponding metabolite was not detected.

**Table S3** The initial content of isoeugenol, eugenol, and vanillin in 10g/L bio-oil

| Compounds | Content (mg/L ) |
| --- | --- |
| Isoeugenol | 68.3±6 |
| Eugenol | 23.5±14.6 |
| Vanillin | 15.9±1.5 |

**Table S4** Comparison of specific activity of monooxygenases from different microbes

| Enzyme | Specific activity  (U/mg) | Orignin | References |
| --- | --- | --- | --- |
| M2 | 30.82±6.1 | *B. pumilus* ZB1 | This study |
| M3 | 26.09±0.5 | *B. pumilus* ZB1 | This study |
| M5 | 29.74±7.79 | *B. pumilus* ZB1 | This study |
| M6 | 52.16±3.77 | *B. pumilus* ZB1 | This study |
| Isoeugenol monooxygenase | 9.2 | *Pseudomonas putida* IE27 | (Yamada et al., 2008) |
| Isoeugenol monooxygenase IEM720 | 4.6 | Metagenome | (Zhao et al., 2018) |
| Isoeugenol monooxygenase | 4.2 | *Pseudomonas nitroreducens* Jin1 | (Ryu et al., 2013) |

**References:**

Ryu, J.-Y., Seo, J., Park, S., Ahn, J.-H., Chong, Y., Sadowsky, M.J., Hur, H.-G., 2013. Characterization of an Isoeugenol Monooxygenase (Iem) from *Pseudomonas nitroreducens* Jin1 That Transforms Isoeugenol to Vanillin. Biosci., Biotechnol., Biochem. 77, 289-294. 10.1271/bbb.120715

Yamada, M., Okada, Y., Yoshida, T., Nagasawa, T., 2008. Vanillin production using *Escherichia coli* cells over-expressing isoeugenol monooxygenase of *Pseudomonas putida*. Biotechnol. Lett 30, 665-670. 10.1007/s10529-007-9602-4

Zhao, L., Xie, Y., Chen, L., Xu, X., Zhao, C.X., Cheng, F., 2018. Efficient biotransformation of isoeugenol to vanillin in recombinant strains of *Escherichia coli* by using engineered isoeugenol monooxygenase and sol-gel chitosan membrane. Process Biochem. 71, 76-81. 10.1016/j.procbio.2018.05.013
